# Treatment of Hypovitaminosis D Is Associated with Improvement in Anemia of Inflammation in Patients with Decompensated Cirrhosis

**DOI:** 10.3390/medsci14020267

**Published:** 2026-05-21

**Authors:** Raquel Diaz-Ruiz, Maria Poca, Eva Roman, Berta Cuyàs, Irene Breton, Rafael Bañares, German Soriano, Rita Garcia-Martinez

**Affiliations:** 1 Department of Digestive Diseases, Instituto de Investigacion Sanitaria, Hospital General Universitario Gregorio Marañon, Universidad Complutense Madrid, 28007 Madrid, Spain; raquel.diaz@salud.madrid.org (R.D.-R.); rbanares@ucm.es (R.B.); 2 Centro de Investigación Biomédica en Red de Enfermedades Hepáticas y Digestivas (CIBERehd), 28029 Madrid, Spain; mpoca@santpau.cat (M.P.); eroman@santpau.cat (E.R.); bcuyas@santpau.cat (B.C.); gsoriano@santpau.cat (G.S.); 3 Department of Gastroenterology, Hospital de la Santa Creu i Sant Pau, Institut de Recerca Sant Pau (IR Sant Pau), Universitat Autònoma de Barcelona, 08041 Barcelona, Spain; 4 Nutrition Unit, Hospital General Universitario Gregorio Marañon, Universidad Complutense Madrid, 28007 Madrid, Spain; irene.breton@salud.madrid.org; 5 Department of Internal Medicine, Instituto de Investigación Sanitaria, Hospital General Universitario Gregorio Marañon, Universidad Complutense Madrid, 28007 Madrid, Spain

**Keywords:** vitamin D, anemia, liver cirrhosis, inflammation, human

## Abstract

**Background/Objectives**: Anemia of inflammation (AI) is a prevalent condition linked to systemic inflammation in several chronic diseases, including chronic liver diseases. Hypovitaminosis D is frequently identified in patients with chronic diseases, and its pathogenic role in anemia is currently under investigation. The aim of this study was to prospectively investigate changes in hemoglobin concentration and inflammatory markers in vitamin D-deficient/-insufficient patients with decompensated cirrhosis after initiating vitamin D supplementation, in addition to the supplementation of other micronutrients if needed. **Methods**: Patients with cirrhosis discharged from decompensation were assessed at baseline and 3 months after vitamin D supplementation. Laboratory parameters of red cell series, nutrition, and micronutrients were assessed in both visits, together with markers of systemic inflammation. **Results**: Thirty-nine patients were included in the study, of whom 33 completed the 3-month evaluation and were analyzed [age: 62.7 ± 10.7 years; gender: n = 29 (87.9%) males; Charlson index: 5.9 ± 1.6; Model for End-Stage Liver Disease (MELD): 12.4 ± 4.5; baseline hemoglobin (Hb): 11.7 ± 1.8 g/dL (anemia n = 24 (72.7%)); mean 25-hydroxyvitamin D (25OHD) plasma level: 15.5 ± 8.6 µg/L]. A significant increase in plasma 25OHD (40.1 ± 17.8, *p* < 0.001) and in Hb (12.4 ± 2.0, *p* = 0.01) was observed at 3 months with a decrease in the prevalence of anemia (n = 17, *p* = 0.015) and of Interleukin 6 in plasma levels [IL-6, 10.7 (5.8–23.3) vs. 6.5 (4.1–11.8), *p* = 0.016]. A greater rise in hemoglobin was correlated with higher plasma IL-6 concentration at baseline. Milder anemia and indexes of hypoferremia at baseline, along with optimal renal function and plasma levels of 25OHD at 3 months, were linked to resolution of anemia. **Conclusions**: Treating vitamin D deficiency together with other micronutrient deficits is associated with inflammation amelioration and improvement in anemia in patients with cirrhosis following discharge from acute decompensation. This paper supports the potential role of vitamin D in the management of anemia in patients with decompensated cirrhosis by modulating systemic inflammation.

## 1. Introduction

Anemia of chronic diseases or anemia of inflammation (AI) is the most common anemia in hospitalized patients and in those who are chronically ill. It has classically been linked to chronic infections, autoimmune diseases, chronic kidney disease and cancer [1]. The spectrum of diseases associated with AI was recently expanded to congestive heart failure [2], chronic obstructive pulmonary diseases [3], obesity, and chronic liver diseases [4], among others. The activation of the systemic inflammatory response results in the production of several cytokines and cell activation. Among these cytokines, Interleukin 6 (IL-6) and Interleukin 1β (IL-1β) are potent inducers of hepcidin, the hormone that regulates iron homeostasis. Hepcidin blocks dietary iron absorption in the duodenum and causes retention of iron in macrophages, resulting in serum hypoferremia. Additionally, several cytokines can impact erythropoiesis by inhibiting the production of erythropoietin [1,5] and also inducing disturbances of iron metabolism [6]. Several micronutrient deficiencies often coexist with systemic inflammation, making it difficult to precisely identify the etiology of anemia and hindering its successful management. Therefore, absolute iron deficiency and cobalamin or folic acid shortage should be identified and treated [5,6]. The role of other micronutrients in anemia is less clear.

Hypovitaminosis D has been found to be prevalent in patients with AI [2,7,8]; however, its pathogenic role remains unclear. Among the extra-skeletal properties of this hormone, its capacity to reduce proinflammatory cytokines has been identified [9]. Moreover, in vitro and vivo studies have shown that vitamin D may act as a suppressor of hepcidin [10,11,12,13]. Also, vitamin D receptor signaling has anti-inflammatory effects and may be effective in downregulating inflammatory processes [6]. Indeed, several observational studies have shown an association between hypovitaminosis D and anemia in different populations [14], particularly those with AI [15]. The ability of micronutrient supplementation other than cobalamin, folate and iron to treat AI in patients with chronic conditions has been poorly evaluated [6,15,16], particularly in patients with liver disease for whom data is absent.

This prospective study investigates the changes in hemoglobin concentration and markers in systemic inflammation in vitamin D-deficient decompensated patients with cirrhosis following discharge from acute decompensation after supplementation.

## 2. Materials and Methods

### 2.1. Design

This observational prospective exploratory study was performed in two tertiary hospitals in Spain (Hospital General Universitario Gregorio Marañon (HGUGM), Madrid, Spain and Hospital Santa Creu i Sant Pau (HSCSP), Barcelona, Spain). Consecutive patients with cirrhosis discharged after an episode of acute decompensation between September 2017 and January 2020 were assessed before initiating vitamin D supplementation, and then at 3 months after enrolment. Other micronutrient deficiencies were also supplemented if identified. Treating vitamin D shortage was a common clinical practice in the participating centers as it was a reasonable recommendation from experts and guidelines, despite the lack of robust evidence. The Institutional Review Board of both hospitals approved the study and all patients signed written consent for participation.

The study was conducted in accordance with the Declaration of Helsinki and approved by the Ethics Committee of Hospital General Universitario Gregorio Maranon (protocol code SUPRAVID on 21 March 2017; number 20170403_193522) and Hospital de la Santa Creu i Sant Pau (26 June 2017; number 17/081). Written informed consent was obtained from all subjects involved in the study.

### 2.2. Patients

Decompensated patients with cirrhosis discharged after hospitalization at both institutions were screened for participation. They were invited to participate if they presented (i) decompensated cirrhosis (ascites, variceal bleeding, hepatic encephalopathy (HE), acute kidney injury—type hepatorenal syndrome); (ii) discharge from liver disease decompensation in the previous 6 weeks; and (iii) age > 18 years. Exclusion criteria were (i) human immunodeficiency virus infection; (ii) hepatocellular carcinoma beyond Milan criteria; (iii) comorbidities with life expectancy < 6 months; (iv) ongoing vitamin D supplementation at the time of evaluation; (v) contraindication for vitamin D supplementation; (vi) neurological invalidating conditions (dementia, cerebrovascular disease, etc.); (vii) previous liver transplantation; (viii) harmful use of alcohol (men: >40 g/d; women: >25 g/d) in the previous 6 months; (ix) cholestatic liver disease; (x) treatment with direct antiviral agents for hepatitis C within the previous 6 months; (xi) Grade 3 acute-on-chronic liver failure; (xii) MELD > 30; and (xiii) immunosuppressive therapy.

Baseline visits were programmed between 1 and 6 weeks after discharge (estimated time for clinical stabilization). Medical records, history of liver disease and medical treatment were logged at baseline and throughout follow-up. Standard blood tests and additional plasma aliquots for further analysis were performed at each visit.

### 2.3. Treatment

Vitamin D was assessed using plasma levels of 25OHD according to local standards. At both sites, 25OHD was measured by chemiluminescent microparticle immunoassay (CMIA) technology fully automated in an Alinity analyzer (Abbott Laboratories, Abbott Park, IL, USA). Those patients with insufficiency (25OHD 20–30 ng/mL or 50–75 nmol/L) or deficiency (25OHD < 20 ng/mL or <50 nmol/L) of vitamin D were supplemented according to the local guidelines available at the time of inclusion and supervised by a nutrition expert in each institution. Briefly, those patients with sufficient vitamin D levels did not receive supplements, those with insufficiency received Calcifediol 0.266 mg (16,000 UI) every two weeks and those with deficiency received 0.266 mg every week with regular controls and readjustments (Supplementary Paragraph S1).

### 2.4. Laboratory Tests

Routine laboratory tests were performed at each visit, comprising hemograms, clot tests, liver and kidney tests and nutritional parameters including micronutrients. In particular, iron status was studied by measuring ferritin and soluble Transferring Receptor (sTfR). Also, subclinical inflammatory markers such as reactive C protein (RCP) and procalcitonin were assessed.

Inflammatory markers: Frozen aliquots of plasma were used for the measurement of the inflammatory mediators IL-12p70 (Interleukin 12), IL-1β (Interleukin 1 beta), TNF-α 2nd gen (Tumor necrosis factor alpha), and IL-6-2nd gen (Interleukin 6), along with vitamin D binding protein (VITDBP) and hepcidin levels. Customized Simple-Plex immunoassay kits (Bio-techne, MN, USA) were used according to the manufacturer’s instructions (https://www.bio-techne.com/reagents/simple-plex-immunoassays/assay-menu?pdfSource=true_neuroinflammation-assay-brochure#human, accessed on 20 March 2026).

### 2.5. Definitions

Anemia: Anemia was defined according to World Health Organization criteria with hemoglobin < 13 g/dL in men and <12 g/dL in women. We used classic diagnostic criteria for defining subtypes of anemia [1,17]:

Iron deficiency anemia: microcytic anemia with low serum ferritin (<100 µg/L) and an sTfR/log ferritin (sTfR/log_10_ferritin) index > 2.

Anemia of inflammation was defined as normocytic with normal or high ferritin (≥100 µg/L) and an sTfR/log ferritin index ≤ 2.

Anemia with both conditions (mixed iron deficiency and inflammation) was defined as low to normal ferritin and an sTfR/log ferritin index > 2.

Given that different etiologies of anemia may coexist, we evaluated and supplemented the etiology of anemia at hospital discharge in the case of iron, folate, or vitamin B12 deficiencies. To clarify the impact of vitamin D on anemia, the initial visit was planned between 1 and 6 weeks following discharge, allowing for clinical and nutritional stabilization.

### 2.6. Statistical Analysis

Continuous variables were reported as mean ± standard deviation or median (inter-quartile range) as appropriate. A Chi-square test or the Fisher exact test were used to study the existence of significant differences between nominal variables. Normality of continuous variables was explored using the Shapiro–Wilk test. Depending on the variable’s distribution, parametric or non-parametric tests were applied to study the differences between groups of patients (Student’s *t*-test, the Mann–Whitney Rank test, or the Kruskal–Wallis test) or between subjects (Paired-samples *t*-test, repeated-measures ANOVA, Wilcoxon test, or Friedman test). Pearson’s or Spearman’s correlation coefficient tests were applied to study correlations between variables. A *p* value < 0.05 was considered statistically significant. The statistical calculations were performed using SPSS 20.0 software (SPSS, Chicago, IL, USA).

## 3. Results

**Figure 1:**
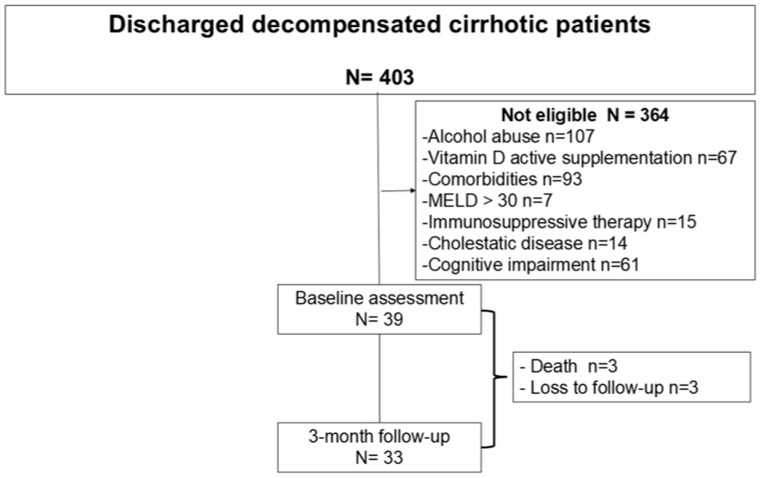
Flowchart of patients screened for participation in the study.

During the inclusion period, 403 patients were evaluated for participation at the time of hospital discharge (Figure 1). Three hundred and sixty-four were not eligible (alcohol abuse n = 107; vitamin D supplementation n = 67; comorbidities n = 93; MELD > 30 n = 7; immunosuppressive therapy n = 15, cholestatic disease n = 14; cognitive impairment n = 61). Thirty-nine patients were ultimately included. Thirty-three patients completed the 3-month evaluation and were analyzed (n = 3 died and n = 3 were lost to follow-up).

### 3.1. Anemia and Vitamin D Status at Baseline

The patients’ baseline characteristics are summarized in Table 1. All subjects had at least one hospitalization the year before admission. The most frequent reasons for decompensation were ascites (n= 30), overt HE (n = 13) and variceal bleeding (n = 10). Regarding vitamin D status, one patient had a sufficient level of vitamin D, nine patients had vitamin D insufficiency and 23 had vitamin D deficiency. The mean baseline 25OHD was 15.5 ± 8.6 µg/L. Notably, 72% of patients (n = 24) had anemia at baseline, of whom eight were iron-deficient, eight were AI and four had mixed etiology (iron deficiency and AI). Importantly, inflammation was involved in 50% of the cases of patients with anemia.

Vitamin D supplementation was initiated at the inclusion visit in all cases with hypovitaminosis. Other micronutrients, if needed, were prescribed 1 to 6 weeks before the baseline visit (while admitted or at hospital discharge, Table 2).

### 3.2. Changes in Red Blood Cells and Vitamin D at 3 Months

At 3 months, a significant increase in plasma levels of vitamin D was observed (15.5 ± 8.6 vs. 40.1 ± 17.8 µg/dL; *p* < 0.001), together with a decrease in PTH (48.4 ± 24.7 vs. 37.0 ± 20.8 ng/L; 0.001). We also found an improvement in several nutritional parameters such as cholesterol, prealbumin, vitamin A and vitamin E. Regarding red cells and iron status, we observed that hemoglobin increased at 3 months (from 11.7 ± 1.8 to 12.4 ± 2.0, *p* = 0.02). Eight patients had resolution of anemia within 3 months, while one patient developed anemia at this point (Table 1).

Regarding the possible underlying etiology of the anemia, micronutrients were assessed and treated if deficiencies were found. As expected, there was a significant change in the prescription of vitamin D and vitamin A. No significant changes in vitamin B12, folate, or iron prescriptions were observed from baseline to 3-month visit (Table 2), nor was there a significant change in plasma concentrations of vitamin B12, folate, ferritin, or transferrin soluble receptor (Table 1). Nevertheless, we observed that eight patients experienced resolution of their anemia, while 16 of them remained anemic. Interestingly, the patients who experienced resolution of anemia included those with iron deficiency anemia, AI, or anemia of mixed origin at baseline. Regarding the inflammatory response, there were no relevant alterations nor significant changes in reactive C protein and procalcitonin. However, we observed a significant decrease in Interleukin 6 from baseline to the 3-month visit (Table 3).

### 3.3. Association Between Anemia, Vitamin D, and Inflammation

**Figure 2:**
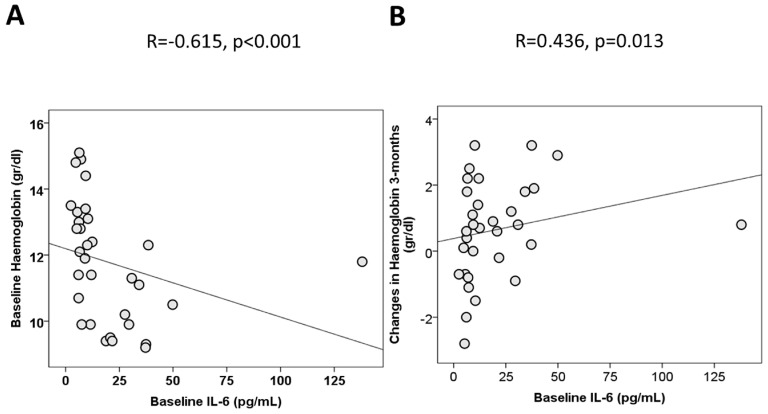
Association between anemia and inflammation. (**A**) shows the correlation between hemoglobin concentration and plasma concentration of IL-6 at baseline. (**B**) shows the baseline IL-6 concentration correlation with improvement in hemoglobin at 3 months.

To find out whether there is a relationship between vitamin D status and anemia of inflammation, we looked for associations at baseline. We observed that lower hemoglobin concentration correlated with markers of malnutrition such as cholesterol (r = 0.437, *p* = 0.011) and zinc (r = 0.406, *p* = 0.019) and with markers of inflammation such as IL-6 (r = −0.615, *p* < 0.001, Figure 2A). In the subgroup of patients with AI or anemia of mixed origin, 25OHD significantly correlated with hemoglobin levels (r = 0.639, *p* = 0.025).

A larger improvement in hemoglobin concentration at 3 months correlated with higher IL-6 at baseline (r = 436, *p* = 0.013, Figure 2B) and improvement in cholesterol (r = 0.442, *p* = 0.011) in the whole cohort. We did not observe a direct association between baseline concentrations of vitamin A or D or changes in those micronutrients with hemoglobin, nor did we find any direct association among those micronutrients and proinflammatory markers. In the subgroup of patients with AI or mixed anemia, we could not identify any association between changes in hemoglobin with baseline or changes in vitamin D, micronutrients, or inflammatory mediators.

To better understand the factors involved in the resolution of anemia, we compared patients who recovered from anemia (n = 8) with those who did not (n = 16; Table 4). The univariate analysis showed that those patients who recovered from anemia had higher hemoglobin and sTfR/log ferritin index at baseline, together with a trend towards higher 25OHD. They also had significantly better renal function and higher 25OHD concentration at 3 months.

## 4. Discussion

This study shows that anemia is highly prevalent in patients with decompensated cirrhosis and vitamin D deficiency discharged from previous hospital admission. In this longitudinal study we observed that treating hypovitaminosis D together with other micronutrients is associated with inflammation amelioration and improvement in anemia. To our knowledge, this is the first study that evaluates the impact of vitamin D supplementation in anemia in patients with decompensated cirrhosis.

Anemia of inflammation has been considered mild to moderate in intensity; however, it may contribute to frailty, falls, cognitive decline, decreased functional ability, and higher mortality risk [18,19].

Treating AI includes the active search and treatment of classical micronutrient deficiencies such as cobalamin, folic acid, and iron, which may coexist [5,6]. In parallel, treating comorbidities such as thyroid dysfunction and optimizing the control of chronic illness is mandatory. However, it is not always possible to eradicate the cause of AI, and this strategy may have limited success in chronic diseases such as heart failure or decompensated cirrhosis. Systemic inflammation of diverse origin is a hallmark of anemia of inflammation, so modulating the inflammatory state may result in additional benefits. However, given that serum hypoferremia in AI is a protective mechanism against microbes, the risk–benefit balance of modulating inflammation can be challenging [18]. Vitamin D has the potential to decrease the synthesis of proinflammatory cytokines [9,20] and to enhance the immune response against microbes [9,21]. Indeed, lower plasma levels of 25OH were found to be associated with severity of infections in several populations [22,23], including patients with cirrhosis [24]. Therefore, vitamin D might hypothetically be useful in treating AI [25,26] without worsening infections [21]. Indeed, it may also be useful in treating and preventing infections, particularly in patients with vitamin D deficiency [27,28].

In this study of patients with decompensated cirrhosis and hypovitaminosis D, we observed that anemia was a prevalent condition (73%) despite ongoing supplementation of cobalamin, folic acid, and iron (Table 2). The etiology of anemia was multifactorial, but inflammation was found to be involved in 50% of cases. Accordingly, lower hemoglobin concentration correlated with higher plasma levels of IL-6, a proinflammatory marker at baseline (Figure 2A). Interestingly, in the subgroup of subjects with anemia of inflammation (50%), lower hemoglobin concentration correlated with the severity of vitamin D deficiency. Micronutrient supplementation, which included vitamin D in all cases, was associated with a decrease in plasma IL-6 concentrations (Table 3). Furthermore, iron deficiency at baseline and higher plasma levels of 25OHD at 3 months were found to be associated with the resolution of anemia. Conversely, impairment in renal function was linked to the persistence of anemia (Table 4). Therefore, our results point towards a resolution of anemia of inflammation reducing systemic IL-6 in previously deficient patients receiving vitamin D supplements.

The potential efficacy of vitamin D supplementation in the resolution of anemia has previously been evaluated in randomized control trials with inconclusive results [15,16]. Those studies were small and conducted in very heterogenous groups, which included healthy populations, highly trained subjects, and participants without anemia or inflammation [15,16]. However, a meta-analysis pointed towards a positive effect in the subgroup of patients with AI treated with vitamin D [16]. The present study included patients with decompensated cirrhosis, a condition characterized by systemic inflammation and immune dysfunction [29], and patients with hypovitaminosis D. The results of our study showing the resolution of anemia following improvement in markers of systemic inflammation are in line with some other reports showing that vitamin D supplementation can partially correct anemia of inflammation [15,16,30], supporting its role in the management of AI in patients with vitamin D shortage. Given that AI is a highly heterogeneous condition, its improvement following vitamin D supplementation may vary depending on the baseline condition and the degree of vitamin D shortage [16,31,32].

This paper has several limitations to be considered. On the one hand, a control group without vitamin D supplementation would be very helpful to better understand whether the presence of anemia and its resolution without supplementation differ from those observed in this cohort. However, it would be ethically controversial to identify decompensated cirrhotic patients with severe vitamin D deficiency and high risk of frailty, infections or other complications, including a high mortality rate, and not prescribe supplements to them [19,33]. Nevertheless, the lack of a control group with sufficient vitamin D or without supplementation constitutes a limitation on the interpretation of our results. On the other hand, information on the activities of vitamin D (25OHD, or activation of vitamin D receptor) would help to better understand the relationship between the vitamin D hormone system and erythropoiesis. Moreover, other supplemented nutrients could influence the observed clinical effects. In fact, lower hemoglobin concentration correlated with markers of malnutrition and inflammation, and a greater increase in hemoglobin was observed in subjects with a higher inflammatory response at the initial visit, but also in those with higher increase in cholesterol during the follow-up. These data support the notion that several pathways (micronutrients and inflammation) are involved in the pathophysiology and resolution of anemia in these patients and that they may coexist. The fact that iron deficiency anemia, AI and anemia of mixed origin may be resolved after three months (Table 1 and Table 4) reinforce these additive effects and support the multifactorial nature of anemia in this population. The implication of these other contributing factors makes it difficult to attribute an observed clinical effect to a specific cause. In this case, it is difficult to quantify the clinical impact of vitamin D alone. Furthermore, the small sample size makes it difficult to have enough statistical power to identify a more robust impact of other potential predictors on anemia resolution. It should also be noted that other relevant groups of patients with decompensated cirrhosis were not included, such as those with a MELD score greater than 30 or grade 3 acute-on-chronic liver failure. This exclusion criterion reduced the loss to follow-up and contributed to a more homogeneous population but also excluded patients with other factors contributing to anemia, such as advanced kidney disease. Therefore, the characteristics of our cohort limit the generalizability of our results, which may differ from those of other larger cohorts or real-world studies. Finally, this study did not include patients with AI associated with chronic diseases other than liver failure. Consequently, these limitations may affect the generalizability of these results.

Despite these shortcomings, we believe that this study provides meaningful clinical information. Measuring vitamin D status is already part of routine clinical practice, and the supplementation of vitamin D deficiency could be a cost-effective strategy in AI with a significant clinical impact. Larger, well-powered trials aiming at investigating the impact of treating hypovitaminosis D in anemia of inflammation and in other relevant clinical outcomes are warranted.

## 5. Conclusions

In summary, this prospective study shows that treating hypovitaminosis D in addition to other micronutrient deficiencies is associated with an improvement in anemia of inflammation in patients with cirrhosis following acute decompensation. Our results suggest a potential role for vitamin D in the treatment of anemia of inflammation and support its action as an immunomodulator.

## Figures and Tables

**Table 1:** Clinical and laboratory characteristics of the decompensated cirrhotic patients included at baseline and at the 3-month visit.

	Baseline N = 33	3 Months N = 33	*p* Value
DEMOGRAPHICS			
Age (years; mean ± SD)	62.7 ± 10.7		
Gender (male; n, %)	29 (87.9%)		
			
ANTHROPOMETRY			
BMI (kg/m^2^; mean ± SD)	28.0 ± 4.8	28.0 ± 5.0	0.895
			
COMORBIDITIES			
Charlson index (mean ± SD)	5.9 ± 1.6	5.9 ± 1.6	0.263
			
CHARACTERISTICS OF CIRRHOSIS			
Alcohol etiology (n, %)	14 (42.4%)		
Esophageal varices (n, %)	27 (81.8%)		
Ascites (n, %)	28 (84.9%)		
SBP (n, %)	8 (24.2%)		
Variceal bleeding (n, %)	15 (45.5%)		
Previous OHE (n, %)	11 (33.3%)		
			
LIVER FUNCTION TEST			
AST (IU/L)	45.3 ± 41.4	40.4 ± 15.7	0.491
ALT (IU/L)	30.9 ± 31.4	28.3 ± 13.6	0.659
GGT (IU/L)	126.1 ± 113	149.8 ± 142.3	0.060
AP (IU/L)	154.3 ± 1.3	150.1 ± 61.0	0.412
CHILD–PUGH (A/B/C; n)	12/17/4		
MELD (mean ± SD)	12.4 ± 4.5	13.0 ± 4.5	0.282
			
LABORATORY TESTS			
Hb (g/dL, mean ± SD)	**11.7 ± 1.8**	**12.4 ± 2.0**	0.01 *
Anemia (n, %)	**24 (72.7%)**	**17 (51.5%)**	0.015 *
Etiology of anemia Iron deficiency/AI/both/others/unknown (n)	**8/8/4/1/3**	8/6/1/1/1 ^†^	
Platelets (1 × 10^9^/L; mean ± SD)	84.9 ± 30.2	80.0 ± 24.1	0.465
INR (mean ± SD)	1.4 ± 0.2	1.4 ± 0.4	0.386
Creatinine (mg/dL; mean ± SD)	0.9 ± 0.3	0.9 ± 0.3	0.824
Na (mEq/L; mean ± SD)	138.2 ± 4.2	138 ± 3.2	0.845
Albumin (g/dL; mean ± SD)	3.5 ± 0.6	3.5 ± 0.5	0.762
Ca (mg/dL; mean ± SD)	9.0 ± 0.6	9.1 ± 0.5	0.753
Phosphate (mg/dL; mean ± SD)	3.3 ± 0.8	3.3 ± 0.5	0.785
Triglycerides (mg/dL, mean ± SD)	83.2 ± 30.4	97.5 ± 65.5	0.279
Cholesterol (mg/dL, mean ± SD)	**125.3 ± 39.3**	**150.1 ± 48.8**	0.003 *
Prealbumin (mg/dL, mean ± SD)	**9.1 ± 4.4**	**10.9 ± 5.0**	0.043 *
Mg (mg/dL; mean ± SD)	1.8 ± 0.2	1.8 ± 0.2	0.713
Zn (µg/L; mean ± SD)	60.3 ± 14.4	66.9 ± 17.1	0.061
Cu (µg/L; mean ± SD)	85.2 ± 29.6	83.3 ± 22.9	0.722
Ferritin (µg/L; mean ± SD)	221.1 ± 264.2	170.3 ± 171.2	0.240
sTfR (mg/L; mean ± SD)	5.0 ± 2.8	4.0 ± 1.9	0.103
sTfR/log ferritin index (median (q1 q3))	2.1 (1.3–3.3)	1.8 (1.1–2.8)	0.696
RCP (mg/dL; mean ± SD)	1.2 ± 1.3	1.3 ± 1.7	0.616
Procalcitonin (µg/L; mean ± SD)	0.11 ± 0.11	0.1 ± 0.1	0.979
25OHD (µg/L; mean ± SD)	**15.5 ± 8.6**	**40.1 ± 17.8**	<0.001 *
25OHD sufficient/insufficient/deficient (n)	**1/9/23**	**22/8/3**	<0.001 *
PTH (ng/L; mean ± SD)	**48.4 ± 24.7**	**37.0 ± 20.8**	0.001 *
Vitamin A (µg/dL; mean ± SD)	**19.5 ± 12.4**	**26.01 ± 14.0**	0.025 *
Vitamin E (µg/dL; mean ± SD)	**1067 ± 404.6**	**1166 ± 389.1**	0.022 *
Vitamin B12 (ng/L; mean ± SD)	750.4 ± 288.9	730.5 ± 407.1	0.784
Folate (µg/L; mean ± SD	11.6 ± 6.6	14.05 ± 6.7	0.096

* Denotes statistical significance. ^†^ Patients who suffered from anemia at 3 months had the following pattern: eight patients had iron deficiency (three of them were iron-deficient at the initial visit and the others developed iron deficiency: two patients with AI at baseline, one patient with mixed etiology, one patient with anemia of unknown origin and one patient without anemia at the beginning of the study); six patients had AI (four of them already had AI at baseline and two developed AI: one patient with iron deficiency anemia and one with anemia of unknown origin). BMI: body mass index; AST: aspartate aminotransferase; ALT: alanine aminotransferase; GGT: gamma glutamyl transferase; AF: alkaline phosphatase; MELD: Model End-Stage Liver Disease; SBP: Spontaneous Bacterial Peritonitis; OHE: overt hepatic encephalopathy; Hb: hemoglobin; INR: international normalized ratio; Na: sodium; Ca: calcium; Mg: magnesium; Zn: zinc; Cu: copper; sTfR: soluble Transferrin receptor; RCP: reactive C protein, 25OHD: 25-hydroxyvitamin D; PTH: parathyroid hormone.

**Table 2:** Summary of active nutritional supplementation at each visit.

	Baseline (n = 33)	3 Months (n = 33)	*p* Value
Vitamin A (N, %)	**16 (50%)**	**23 (69.7%)**	0.0059 *
Vitamin E (N, %)	5 (15.6%)	5 (15.6%)	1.000
Vitamin D (N, %)	**0 (0%)**	**31/32 (96.8%)**	<0.0001 *
Vitamin K (N, %)	3 (9.4%)	5 (15.6%)	0.7078
Vitamin B12 (N, %)	2 (6.25%)	1 (3.13)	1.000
Folic acid (N, %)	5 (15.6%)	6 (18.75%)	1.000
Calcium (N, %)	2 (6.25%)	2 (6.25%)	1.000
Magnesium (N, %)	3 (9.4%)	2 (6.25%)	1.000
Iron (N, %)	7 (21.9%)	9 (28.1%)	0.3333
Hypercaloric oral nutritional supplement (N, %)	2 (6.25%)	2 (6.25%)	1.000

* Denotes statistical significance. Comment: At hospital discharge, the identified nutritional deficiencies were supplemented according to local practice. Additionally, if the patient was eligible, the/shey were then called for an appointment within 1 to 6 weeks after discharge for initial assessment and vitamin D supplementation. This may explain the fact that at baseline evaluation, 50% of patients were receiving vitamin A, 21% were receiving iron, 15% were on folic acid supplementation and 9.4% were receiving magnesium, among other supplements. As expected, a significant change in prescription was observed in vitamin D supplementation. It should be noted that 69.7% of patients received vitamin A supplementation at 3 months.

**Table 3:** Assessment of inflammation at baseline and 3 months after inclusion.

	Baseline (n = 33)	3 Months (n = 33)	*p* Value
IL12p70 (pg/mL median (q1 q3))	2.7 (1.1–3.5)	1.7 (0.7–3.3)	0.331
IL-1β (pg/mL; median (q1 q3))	0.5 (0.3–0.8)	0.4 (0.1–0.8)	0.650
TNF-α (pg/mL; median (q1 q3))	11.9 (8.7–15.7)	11.2 (8.8–14.7)	0.887
IL-6 (pg/mL; median (q1 q3))	**10.7 (5.8–23.3)**	**6.5 (4.1–11.8)**	0.016 *
Hepcidin (pg/mL; median (q1 q3))	26,354 (7674–68,439)	20,408 (6118–85,931)	0.730
VitDBP (mg/mL; median (q1 q3))	116.5 (34.6–166.3)	94.6 (48.8–126.0)	0.331

* Denotes statistical significance. IL12p70: Interleukin 12; IL-1β: Interleukin 1 beta; TNF-α: Tumor necrosis factor alpha; IL-6: Interleukin 6; VITDBP: vitamin D binding protein.

**Table 4:** Factors related to the resolution of anemia at 3 months.

Factors	Reversed Anemia N = 8	Persistent Anemia N = 16	*p* Value
Age (years; mean ± SD)	61.5 ± 14.5	64.8 ± 8.6	0.566
BMI (kg/m^2^; mean ± SD)	27.0 ± 3.0	20.0 ± 5.5	0.364
Charlson index (mean ± SD)	5.9 ± 1.6	6.3 ± 1.7	0.554
Alcohol etiology (n, %)			
CHILD–PUGH (A/B/C; n)			0.742
MELD (mean ± SD)	13.4 ± 4.7	12.9 ± 4.3	0.821
**BASELINE LABORATORY**			
Hb (g/dL, mean ± SD)	**11.6 ± 0.9**	**10.5 ± 1.2**	**0.032**
Etiology of anemia at baseline Iron deficiency/AI/both/others/unknown (n)	4/2/1/1/0	4/6/3/1/2	0.661
INR (mean ± SD)	1.4 ± 0.2	1.4 ± 0.2	0.497
Creatinine (mg/dL; mean ± SD)	0.8 ± 0.1	1.0 ± 0.4	0.254
Na (mEq/L; mean ± SD)	136.8 ± 4.6	139 ± 5.0	0.172
Albumin (g/dL; mean ± SD)	3.4 ± 0.6	3.2 ± 0.5	0.347
Ca (mg/dL; mean ± SD)	8.9 ± 0.6	8.9 ± 0.5	0.846
Phosphate (mg/dL; mean ± SD)	3.4 ± 0.6	3.1 ± 0.8	0.540
Triglycerides (mg/dL, mean ± SD)	75.7 ± 28	76.6 ± 21	0.932
Cholesterol (mg/dL, mean ± SD)	114.1 ± 41	113.1 ± 26.2	0.950
Prealbumin (mg/dL, mean ± SD)	8.4 ± 3.1	8.1 ± 3.9	0.875
Mg (mg/dL; mean ± SD)	1.8 ± 0.2	1.8 ± 0.3	0.794
Zn (µg/L; mean ± SD)	53.9 ± 13.4	56.9 ± 13.0	0.603
Cu (µg/L; mean ± SD)	101.0 ± 35.8	79.9 ± 24.2	0.101
Ferritin (µg/L; mean ± SD)	241.0 ± 368	188.6 ± 176.1	0.714
sTfR (mg/L; mean ± SD)	7.7 ± 4.0	4.4 ± 2.1	0.055
sTfR/log ferritin index (median (q1 q3))	**3.3 (2.2–9.0)**	**1.9 (1.1–2.6)**	**0.033**
RCP (mg/dL; mean ± SD)	1.5 ± 2.0	1.4 ± 1.2	0.830
Procalcitonin (µg/L; mean ± SD)	0.15 ± 0.1	0.11 ± 0.11	0.414
25OHD (µg/L; mean ± SD)	17.7 ± 6.5	12.8 ± 9.5	0.052
PTH (ng/L; mean ± SD)	34.7 ± 6.9	55.5 ± 26.7	0.357
Vitamin A (µg/dL; mean ± SD)	17.3 ± 8.0	15.2 ± 12.0	0.291
Vitamin E (µg/dL; mean ± SD)	996.0 ± 258.3	910.2 ± 296.1	0.494
Vitamin B12 (ng/L; mean ± SD)	850.2 ± 371.8	738.4 ± 288.7	0.500
Folate (µg/L; mean ± SD	7.9 ± 5.9	11.8 ± 6.7	0.185
IL12p70 (pg/mL median (q1 q3))	1.8 (0.9–2.8)	2.7(1.2–3.8)	0.482
IL-1β (pg/mL; median (q1 q3))	0.52 (0.4–0.9)	0.5 (0.3–0.9)	0.681
TNF-α (pg/mL; median (q1 q3))	15.1 (12.3–18.1)	13.2 (8.7–20.8)	0.466
IL-6 (pg/mL; median (q1 q3))	11.1 (7.9–32.0)	21.7 (6.9–34.2)	0.681
Hepcidin (pg/mL; median (q1 q3))	5475 (970–50,459)	14,644 (1796–352,333)	0.428
VitDBP (mg/mL; median (q1 q3))	108 (76.6–169.5)	76.8 (28.4–114)	0.149
**LABORATORY 3 MONTHS**			
Creatinine (mg/dL; mean ± SD)	**0.8 ± 0.2**	**1.0 ± 0.4**	**0.023**
Ferritin (µg/L; mean ± SD)	154 ± 104	183 ± 212	0.535
sTfR (mg/L; mean ± SD)	3.8 ± 1.1	4.4 ± 2.4	0.731
sTfR/log ferritin index (median (q1 q3))	1.4 (1.7–2.3)	2.0 (1.0–4.3)	0.802
25OHD (µg/L; mean ± SD)	**52.3 ± 15.7**	**35.7 ± 14.3**	**0.028**
PTH (ng/L; mean ± SD)	31.1 ± 13.7	40.6 ± 25.9	0.547
IL12p70 (pg/mL median (q1 q3))	1.9 (0.8–3.9)	2.1 (0.5–4.3)	0.914
IL-1β (pg/mL; median (q1 q3))	0.4 (0.3–0.5)	0.4 (0.0–0.8)	0.788
TNF-α (pg/mL; median (q1 q3))	13.0 (10.0–15.4)	9.9 (3.5–16.7)	0.527
IL-6 (pg/mL; median (q1 q3))	9.2 (5.8–15.3)	8.7(3.3–16.1)	0.762
Hepcidin (pg/mL; median (q1 q3))	20,408 (8040–29,667)	5442 (1029–111,495)	0.412
VitDBP (mg/mL; median (q1 q3))	98.5 (60.7–102.0)	47.9 (29.4–78.5)	0.164

BMI: body mass index; MELD: Model End-Stage Liver Disease; INR: international normalized ratio; Na: sodium; Ca: calcium; Mg: magnesium; Zn: zinc; Cu: copper; sTfR: soluble transferrin receptor; RCP: reactive C protein; 25OHD: 25-hydroxyvitamin D; IL12p70: Interleukin 12; IL-1β: Interleukin 1 beta; TNF-α: Tumor necrosis factor alpha; IL-6: Interleukin 6; VITDBP: vitamin D binding protein.

## Data Availability

The original contributions presented in this study are included in the article/Supplementary Materials. Further inquiries can be directed to the corresponding author.

## Supplementary Materials

The following supporting information can be downloaded at https://www.mdpi.com/article/10.3390/medsci14020267/s1, Supplementary Paragraph S1: Supplementation of vitamin D according to baseline 25OHD. Table S1: The IOM and ES definitions of vitamin D deficiency (25OHD).

## Abbreviations

The following abbreviations are used in this manuscript:

AI	Anemia of inflammation
Hb	Hemoglobin
HE	Hepatic Encephalopathy
IL-1β	Interleukin 1 beta
IL12p70	Interleukin 12
IL-6	Interleukin 6
MELD	Model End-Stage Liver Disease
RCP	Reactive C protein
SBP	Spontaneous Bacterial Peritonitis
sTfR	soluble Transferring Receptor
TNF-α	Tumor necrosis factor alpha
VITDBP	Vitamin D binding protein
25OHD	25-hydroxyvitamin D
