# Supplementary material for: Treatment of Hypovitaminosis D Is Associated with Improvement in Anemia of Inflammation in Patients with Decompensated Cirrhosis
Source: Med Sci (Basel). 2026 May 21;14(2):267. doi: 10.3390/medsci14020267 (PMC13214777; doi:10.3390/medsci14020267)
Supplement: Supplementary file 1 [file medsci-14-00267-s001.zip › medsci-4242374-supplementary.pdf]

1. **Supplementary Paragraph S1: Supplementation of vitamin D according to baseline 25OHD.**

1.1. Definition: Vitamin D is a fat-soluble vitamin that exhibits hormonal functions. It can be obtained from a small number of foods, and it is mostly activated in the skin with sun exposure. The most commonly vitamin D metabolite used by clinicians and researchers to indicate vitamin D status is 2OHD. However, taking a single indicator to evaluate these complex interactions has its shortcomings. Besides, when this research was conducted, there was no globally accepted cut-offs for defining vitamin D deficiency and insufficiency. The most common internationally adopted cut-offs for vitamin D deficiency/insufficiency are those proposed by Endocrine Society (ES) and Institute of Medicine (IOM). The practical and clinically accepted cut-off levels for vitamin D adequacy and insufficiency are based on the regulatory mechanisms along the calcium, parathyroid hormone and vitamin D axis.

Table S1. The IOM and ES definitions of vitamin D deficiency (25OHD).

	Sufficiency	Insufficiency	Deficiency
IOM	20-30 ng/mL<br>50-75 nmol/mL	12-20 ng/ml<br>30-50 nmol/L	<12 ng/mL<br><30 nmol/mL
ES	30-100 ng/mL<br>75-250 nmol/mL	20-29 ng/mL<br>50-75 nmol/mL	<20 ng/mL<br><50 nmol/ml

1.2. Recommendations:

The supplementary recommendations are different considering Endocrine Society (ES) and Institute of Medicine (IOM). These differences are mainly based on the population reference for supplementation.

	Reference population	RECOMENDED DOSE	MAXIMUN DAILY DOSE
IOM	Healthy population<br>(low expected benefit)	600-800 UI/day	4000 UI/day
ES	Patients with osteoporosis and other risk conditions (higher objective and doses)	If deficiency:<br>50000 UI/week (8 weeks).<br>Maintenance 1500-2000 UI/day.	4000 UI/day

1.3. Common local clinical practice:

BASELINE VIT D STATUS	INITIAL DOSE	MAINTENANCE	CONTROL	OBJECTIVE
INSUFFICIENCY<br>25OHD 20-30 ng/mL	Vitamin D 16000UI/2 weeks	Vitamin D 16000UI /2 weeks	3 months	25OHD<br>30-50 ng/mL<br>Ideally 40ng/mL
DEFICIENCY<br>25OHD <20 ng/mL	Vitamin D 16000UI<br>/1 week	Vitamin D 16000UI /1 week	3 months	25OHD<br>30-50 ng/mL<br>Ideally 40ng/mL

a) Dose and maintenance were individualized in patients with special characteristics such as severe deficiency, malabsorption or suboptimal response.

b) Expected increases in serum 25OHD according to supplementation:
100 UI/d increase serum 25OHD 1-2 ng/mL.
1000UI/d increase serum 25OHD 10-20 ng/mL.

2000UI/d increase serum 20-40 ng/mL.

c) Given that plasma measurements are not perfect, the plasma 25OHD target following supplementation is 40 ng/mL (which ensures adequate vitamin repletion remaining within the normal range).
d) Calcium, Phosphate and 25OHD were monitored during the study.
e) Dose adjustments during follow-up:
   - If 25OHD < 60 ng/mL: Keep same dose (16000UI /1 week if baseline deficiency and 16000UI/2 weeks if insufficiency)
   - IF5OHD $\geq$ 60 ng/mL: decrease dose to half of initial dose (16000UI /2 weeks if deficiency and 16000UI/4 weeks if insufficiency).
